# Validation tests for cryo-EM maps using an independent particle set

**DOI:** 10.1016/j.yjsbx.2020.100032

**Published:** 2020-07-21

**Authors:** Sebastian Ortiz, Luka Stanisic, Boris A Rodriguez, Markus Rampp, Gerhard Hummer, Pilar Cossio

**Affiliations:** a Biophysics of Tropical Diseases, Max Planck Tandem Group, University of Antioquia UdeA, Calle 70 No. 52-21, Medellín, Colombia; b Max Planck Computing and Data Facility, 85748 Garching, Germany; c Grupo de Fósica Atómica y Molecular, Instituto de Física, Facultad de Ciencias Exactas y Naturales, Universidad de Antioquia UdeA, Calle 70 No. 52-21, Medellín, Colombia; d Department of Theoretical Biophysics, Max Planck Institute of Biophysics, 60438 Frankfurt am Main, Germany; e Institute of Biophysics, Goethe University, 60438 Frankfurt am Main, Germany

**Keywords:** Cryo-EM, Validation, Reconstruction, 3D refinement, Independent, Raw data, BioEM

## Abstract

Cryo-electron microscopy (cryo-EM) has revolutionized structural biology by providing 3D density maps of biomolecules at near-atomic resolution. However, map validation is still an open issue. Despite several efforts from the community, it is possible to overfit 3D maps to noisy data. Here, we develop a novel methodology that uses a small independent particle set (not used during the 3D refinement) to validate the maps. The main idea is to monitor how the map probability evolves over the control set during the 3D refinement. The method is complementary to the gold-standard procedure, which generates two reconstructions at each iteration. We low-pass filter the two reconstructions for different frequency cutoffs, and we calculate the probability of each filtered map given the control set. For high-quality maps, the probability should increase as a function of the frequency cutoff and the refinement iteration. We also compute the similarity between the densities of probability distributions of the two reconstructions. As higher frequencies are included, the distributions become more dissimilar. We optimized the BioEM package to perform these calculations, and tested it over systems ranging from quality data to pure noise. Our results show that with our methodology, it possible to discriminate datasets that are constructed from noise particles. We conclude that validation against a control particle set provides a powerful tool to assess the quality of cryo-EM maps.

## Highlights

• Use of an independent particle set to validate 3D density maps from cryo-EM.
• The posterior probability of the map should increase as a function of the iteration and low-pass frequency cutoff.
• The similarity between the distributions of the posterior probabilities is an indicator of the map quality.
• A control particle set provides valuable information for cryo-EM map validation.

## Graphical abstract

**Figure:**
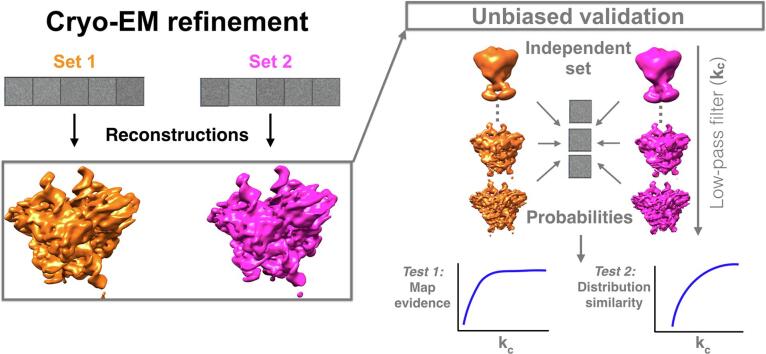

## Introduction

Cryo-electron microscopy (cryo-EM) has revolutionized structural biology by providing electron density maps of biomolecules that were difficult to resolve with X-ray crystallography or nuclear magnetic resonance (Kühlbrandt, 2014, Cheng, 2015, Murata and Wolf, 2018). The introduction of direct electron detection cameras (Wu et al., 2016, McMullan et al., 2016) and novel computational algorithms (Kervrann et al., 2016, Cossio and Hummer, 2018) has enabled the reconstruction of density maps with near-atomic details. To date, an exponential-growing number of maps, and their corresponding atomic models (Afonine et al., 2018), are being deposited in the electron microscopy (Lawson et al., 2011) and protein data banks (Berman et al., 2000) (EMDB and PDB, respectively).

Typically, cryo-EM maps are reconstructed using the gold-standard procedure (Henderson et al., 2012, Scheres and Chen, 2012). The particles are divided into two sets, and two independent reconstructions are generated. The reconstructions are refined iteratively using maximum-likelihood (Sorzano et al., 2004, Tang et al., 2007) or Bayesian techniques (Scheres, 2012, Punjani et al., 2017). At each iteration, the Fourier Shell Correlation (FSC) (Saxton and Baumeister, 1982, Harauz and van Heel, 1986) between the independent reconstructions is computed. Fixed FSC threshold criteria at 0.143 (Rosenthal and Rubinstein, 2015) or 0.5 (Harauz and van Heel, 1986) are used to determine the resolution of the reconstructions (*i.e.*, the size of the smallest reliable detail). The refinement process is halted when the resolution of the reconstructions stops improving. In the end, the maps are masked, sharpened, and a final resolution is determined.

However, despite several efforts from the cryo-EM community, map validation is still problematic. In the recent Map Challenge, it has been shown that there is no absolute ‘gold standard’ (Heymann, 2018). The protocols are user-dependent, and there can be biases due to processing workflows. For instance, in the FSC calculation, the resolution estimate is dependent on the radius of the shell in Fourier space, and on the point symmetry of the biomolecule (Van Heel and Schatz, 2005, Sorzano et al., 2017). The use of a fixed threshold for the FSC is restricted by the assumption that the noise and the signal are orthogonal (Van Heel and Schatz, 2005). In addition, the mask can be a source for overestimating the resolution (Penczek, 2010, Pintilie et al., 2016, Rosenthal and Rubinstein, 2015). Therefore, the best criteria to estimate the map resolution are still debated in the cryo-EM community (Sorzano et al., 2017). For example, inconsistencies when reporting the resolution have been highlighted in Ref. (Afonine et al., 2018), where the values of the resolution reported in the model (from the PDB) and in the map (from the EMDB) were different for nearly one-third of the deposited data. Moreover, it has been found that more than 70% of the maps in the EMDB have moderate to low agreement with the model, mostly because of the limited resolvable features of the maps (Neumann et al., 2018). In extreme cases, maps can be reconstructed from pure-noise images (Henderson, 2013, Shatsky et al., 2009). These issues can lead to cryo-EM maps built from aligned noise.

Therefore, methods that validate the quality of the maps and models are fundamental for cryo-EM. Several methods use the FSC curve to assess the quality of the reconstructions. By discarding shells from the reference map used for the alignment, Shaikh et al. (Shaikh et al., 2003) encountered a different behaviour of the FSC curve between reconstructions that were made from pure-noise images or real particles. Signatures of overfitting can be detected by randomizing the phases beyond a certain frequency (Scheres and Chen, 2012, Chen et al., 2013), for non-overfitted maps, the FSC should drop close to zero after that frequency. Estimates of the map resolution, which take into account the symmetry of the molecule and the non-orthogonality of the signal and noise, are obtained with the 1/2 bit non-fixed FSC threshold (Van Heel and Schatz, 2005, Afanasyev et al., 2017). The local resolution in a map can be evaluated using the background noise of the reconstruction (Kucukelbir et al., 2014) or by masking different regions with the FSC (Cardone et al., 2013, Pintilie et al., 2016). For other methods, the particle alignment provides quality indicators of the reconstruction, for example, using tilt-pair analysis (Rosenthal, 2016, Rosenthal and Rubinstein, 2015, Rosenthal and Henderson, 2003, Henderson et al., 2011, Penczek et al., 1994) or by assessing the reproducibility of the orientation assignment (Vargas et al., 2017, Vargas et al., 2016). Moreover, several metrics that monitor cross-correlations in real or Fourier space between the maps and models indicate the reliability of the resolution (Afonine et al., 2018, Neumann et al., 2018, Brown et al., 2015). Recently, deep learning algorithms have been introduced to automatically classify maps into high, medium, and low resolution (Avramov et al., 2019). These methods have the limitation that they do not use the raw data, which ultimately come from the individual particles, but they only use the maps or models that are the product of processing and averaging.

In comparison to the widely used cross-validation methods for X-ray crystallography (Brünger, 1992) and nuclear magnetic resonance (Brünger et al., 1993, Clore and Garrett, 1999), there are few methods available for cryo-EM (Shaikh et al., 2003, Falkner and Schroeder, 2013). Heymann (Heymann, 2015) showed that reconstructions from sets of real particles have higher resolutions than reconstructions from pure-noise particles, which can be used as a consistency test of the data. However, this test requires processing and averaging the particles for generating the reconstructions and extracting the resolutions.

Here, we propose an unbiased strategy that validates cryo-EM reconstructions using a small control set of particles that are omitted from the refinement process. We do not focus on determining a specific value for the resolution, but the main idea is to monitor how the performance of the reconstructions evolves during the refinement procedure over unbiased and independent data. We first calculate the Bayesian inference of electron microscopy (BioEM) (Cossio and Hummer, 2013, Cossio et al., 2017) probability of the maps, given the set of independent particles, as a function of a low-pass frequency cutoff of the reconstructions. High-quality maps should increase in probability for higher frequency cutoffs and higher refinement iterations. We then show that the similarity between the probability distributions of the two reconstructions from the gold-standard procedure is an additional quality indicator. Finally, we test the method on different systems and asses its effectiveness to discriminate high quality maps, which are reconstructed from true signal instead of noise.

## Methods

### Benchmark systems

We used the following benchmarks that represent diverse biomolecular families and cryo-EM systems:

*The human hyperpolarization-activated cyclic nucleotide-gated channel* (HCN1) is a voltage-dependent ion channel, which was resolved to high resolution using cryo-EM (Lee and MacKinnon, 2017). The system was resolved in two conformational states, an *apo* state and a cAMP-bound state, to ~3.5 Åusing RELION 3D-refinement (Scheres, 2012). 55870 particles belonging to the *apo* state together with the defocus information are available in the Electron Microscopy Public Image Archive (EMPIAR) (Iudin et al., 2016) with code 10081.

*The recombination-activating genes RAG1-RAG2* form a complex (RAG1-RAG2) that plays an essential role in the generation of antibodies and antigen-receptor genes in a process called V(D) J recombination. Two main structures of the RAG1-RAG2 complex can be distinguished during the V(D) J recombination, a synaptic paired complex and the signal end complex (SEC). These states were resolved to 3.7 and 3.4 Å, respectively, using cryo-EM (Ru and Others, 2015). 81946 processed picked particles from the SEC state are deposited in the EMPIAR data bank with code 10049. The defocus information is available for these particles.

*The mammalian transient receptor potential TRPV1* ion channel (TRPV1) is the receptor for capsaicin. Its structure was determined to 3.4 Åusing cryo-EM (Maofu et al., 2013). A set of 35645 processed particles for this system are found in the EMPIAR data bank with code 10005. The defocus information is also available for these particles.

*The human immunodeficiency virus type 1 envelope glycoprotein trimer* (HIV-ET) is a membrane-fusing machine which mediates virus entry into host cells. The structure of the apo HIV-1 envelope glycoprotein in the trimer-conformation was determined to 6 Åusing the 0.5 FSC threshold with cryo-EM (Mao et al., 2013). A set of 124478 particles used in the refinement process is available in EMPIAR with code 10008. The defocus information is also available for these particles.

For all of the above cases, a subset of 5000 particles was randomly selected to be used as the control set. Specifically, these particles are not used in the refinement processes.

*Pure-noise images:* we generated a set of synthetic 1000 pure-noise particles. Each particle contains random intensities following a Gaussian distribution with zero mean and unit variance (for details see the Supplementary Information). These images were used as a “false” control set to assess the RAG1-RAG2 reconstructions.

### 3D refinement

The RELION (Scheres, 2012) software was used to reconstruct the cryo-EM maps. For all systems, we assume that the deposited particles correspond to the same state. Therefore, the preprocessing steps of 2D or 3D classification are not performed. As the initial reference map for the 3D refinement, we use the final map reported by the authors low-pass filtered to 60 Å. This was done to minimize the risk of model bias (Scheres and Chen, 2012). The 3D-refinement procedure implements the gold-standard approach by splitting the data into two random halves (sets i=1,2) and performing two independent reconstructions. We note that the number of particles used for these reconstructions was slightly less than those of the original works because the particles from the control set were taken out. In all cases, we used the RELION default parameters, and point-group symmetries reported by the authors. Table 1 summarizes the results obtained from the 3D refinement. The resolutions are in accordance with the reported ones, taking into account that the post-processing steps were not performed, and that the control set of particles was excluded from the refinement.

**Table 1:** Summary of the results from the 3D-refinement using RELION (Scheres, 2012) for the cryo-EM systems.

System	#Particles	Symmetry	#iterations	Final resolution∗
HCN1	50870	C4	17	4.2 Å
RAG1-RAG2	79946	C2	21	3.8 Å
TRPV1	30645	C4	24	5.3 Å
HIV-ET	119478	C3	10	9.9 Å

∗ using the 0.143 FSC threshold.

### Low-pass filtering

Consider a map *m* generated from an iteration of the 3D refinement. Let $F_{m}(k)$ be its 3D-Fourier transform, where k is the reciprocal vector. We perform a low-pass filter on the map, $F_{m}^{k_{c}}(k)$, up to a frequency cutoff $k_{c}$. The resulting filtered map is

$$F_{m}^{k_{c}}(k)=\left\{\begin{array}{cc} F_{m}(k) & k⩽k_{c}\\ 0 & \mathrm{otherwise} \end{array}\right)$$

We use the code *lowpassmap*_*fftw* available from the Rubinstein lab webpage (Rubinstein, xxxx) to perform this calculation. We then convert the map into real space by applying the inverse Fourier transform of $F_{m}^{k_{c}}(k)$. The real-space filtered map is masked and then used as input for the BioEM computation (see below).

### BioEM posterior probabilities

The BioEM method (Cossio and Hummer, 2013) uses a Bayesian framework to quantify the consistency between an experimental image ω and a given map *m* (or model) by calculating a posterior probability $P_{m\omega }$. BioEM takes into account the relevant physical parameters (Θ) for the image formation: center displacement, normalization, offset, noise, orientation and CTF parameters (defocus, amplitude, and B-factor). $P_{m\omega }$ is calculated by integrating-out all parameters

(1) $$P_{m\omega }\propto \int L(\omega |\Theta ,m)p(\Theta )d\Theta ,$$

where p(Θ) are the prior probabilities parameters and L(ω∣Θ,m) is the likelihood function. For ensemble determinations, the posterior can be multiplied by the prior probabilities of each model, as described in Ref. Cossio and Hummer (2013). We considered the prior probabilities of maps, orientations and center displacement uniform over the integration intervals. Gaussian priors for the CTF parameters were used similarly as in Ref. Cossio et al. (2018). In Eq. (1), the integrals over the offset, noise and normalization are performed analytically (Cossio and Hummer, 2013), and that over the center displacement is described in Ref. Cossio et al. (2017). The integral over the orientations and CTF defocus is done using a double-round algorithm, which is described in the following subsection.

Similarly as in Ref. Cossio and Hummer (2013), we define a noise model $P_{\mathrm{Noise}}={\left(2\pi \lambda^{2}e\right)}^{-N_{\mathrm{pix}}/2}$ where $N_{\mathrm{pix}}$ is the number of pixels and λ is the image variance (by default λ=1). $P_{\mathrm{Noise}}$ is used as a reference to compare the posterior probabilities to a hypothetical model composed of Gaussian noise of zero mean and unit variance. We note that $P_{\mathrm{Noise}}$ is a constant for each system, and $\ln(P_{m\omega }/P_{\mathrm{Noise}}$) estimates how many log-units the map is more probable than the Gaussian noise model for particle ω.

### BioEM algorithm

To optimize the computations, we divided the BioEM posterior calculation into two rounds. The objective of the first round is to obtain the best orientations for each particle. In this round, an all-orientations to all-particles algorithm is performed (Cossio et al., 2017). As the BioEM input map, we used the final reconstruction from the refinement with a mask and without low-pass filtering. To sample the orientations, we used 36864 quaternions that sample uniformly orientation space (Yershova et al., 2010). The particles were grouped into sets with similar experimental defocus with 0.4μm range, and an independent orientation search was performed for each group. In this round, the best 10 orientations for each particle are extracted. An example of the BioEM input for the first round is presented in the Supplementary Information.

Starting in round 2, we calculate the BioEM posterior probability using a fixed list of quaternions and the experimental defocus for each particle, for all low-pass filtered reconstructions from the refinement iterations. The list of quaternions contains 1250 orientations concentrated around on the 10 best orientations for each particle from BioEM round 1. Therefore, the optimal orientations and translations are determined at every iteration for every low-passed filtered map. However, we note that the space of possible angles is not the entire SO3 but it is the reduced list of 1250 quaternions determined only once for each particle in round 1. This procedure is described in detail in Ref. Cossio et al. (2018).

In round 2, we used 8 filtering-frequencies for each reconstruction; these were distributed uniformly from $1/(p_{s}\sqrt{N_{\mathrm{pix}}})$ to $1/(3p_{s})$ where $p_{s}$ is the pixel size. If one wants to analyze the behavior at very low-resolution or very high-resolution, the frequency-cutoff range should be modified (*e.g.* as in Supplementary Fig.1). All reconstructions were masked using the same mask as for round 1. An example of the BioEM input file for round 2 is presented in the Supplementary Information.

### BioEM code

The BioEM code has been extended with several optimizations, which drastically increase performance for the second round of calculations. Most importantly, the main code structure and algorithm were modified to allow for a parallel comparison of multiple orientations to a single particle image. Initial reading of the input files has been parallelized, and the overall memory consumption decreased. These code changes lead to more efficient utilization of the computing resources, and hence to a faster calculation of posterior probabilities, especially for the workloads specific to the second round. For more information, we refer the reader to the BioEM user manual: https://readthedocs.org/projects/bioem/.

### Normalized Jensen-Shannon divergence

Measuring a distance among probability distributions is a common task in statistics. Most measures include concepts from information theory, such as the Kullback–Leibler divergence (Kullback, 1968, Lin, 1991) or the Shannon entropy (Cover and Thomas, 2006). In this work, we measure the statistical similarity between the probability distributions from reconstructions from set 1 and set 2 calculated over the control set. We define a metric that is the Jensen-Shannon divergence (Cover and Thomas, 2006, Lin, 1991) normalized by the individual Shannon entropies

(2) $$\mathrm{NJSD}=\frac{\sum_{\omega }[\ln(2)+P_{1\omega }\ln(P_{1\omega })+P_{2\omega }\ln(P_{2\omega })]}{2{\left(\sum_{\omega }P_{1\omega }\ln(P_{1\omega })\sum_{\omega }P_{2\omega }\ln(P_{2\omega })\right)}^{1/2}},$$

where $P_{1\omega }$ and $P_{2\omega }$ are the probabilities of the reconstructions from set 1 and 2, respectively. $P_{1\omega }$ and $P_{2\omega }$ are not normalized in the BioEM calculation, therefore, to calculate Eq. (2), we enforce the simple normalization $P_{1\omega }+P_{2\omega }=1$ for each image ω, frequency cutoff and iteration. We note that to enforce this normalization both probabilities from set 1 and 2 are required. For simplicity of notation, we have omitted the dependency of the probabilities on the frequency cutoff $k_{c}$.

In Eq. (2), the numerator measures the similarity between the densities of probability distributions, and the Shannon entropies in the denominator play the role of a normalization factor. Some important properties of the NJSD metric are that it is positive, symmetric and its lower bound is 0 if and only if $P_{1\omega }=P_{2\omega }$ for all particles ω.

## Results and discussion

### Validation protocol using an independent particle set

We propose a statistical framework for the validation of cryo-EM reconstructions. The validation analysis is done over a small control set of particles not used in the refinement process. Our main objective is to assess how well the maps perform over unseen data, which is the core of cross-validation. Therefore, having an independent set guarantees that the estimate of the quality of the reconstructions is free of biases from the optimization of the target function used in the refinement.

**Fig. 1:**
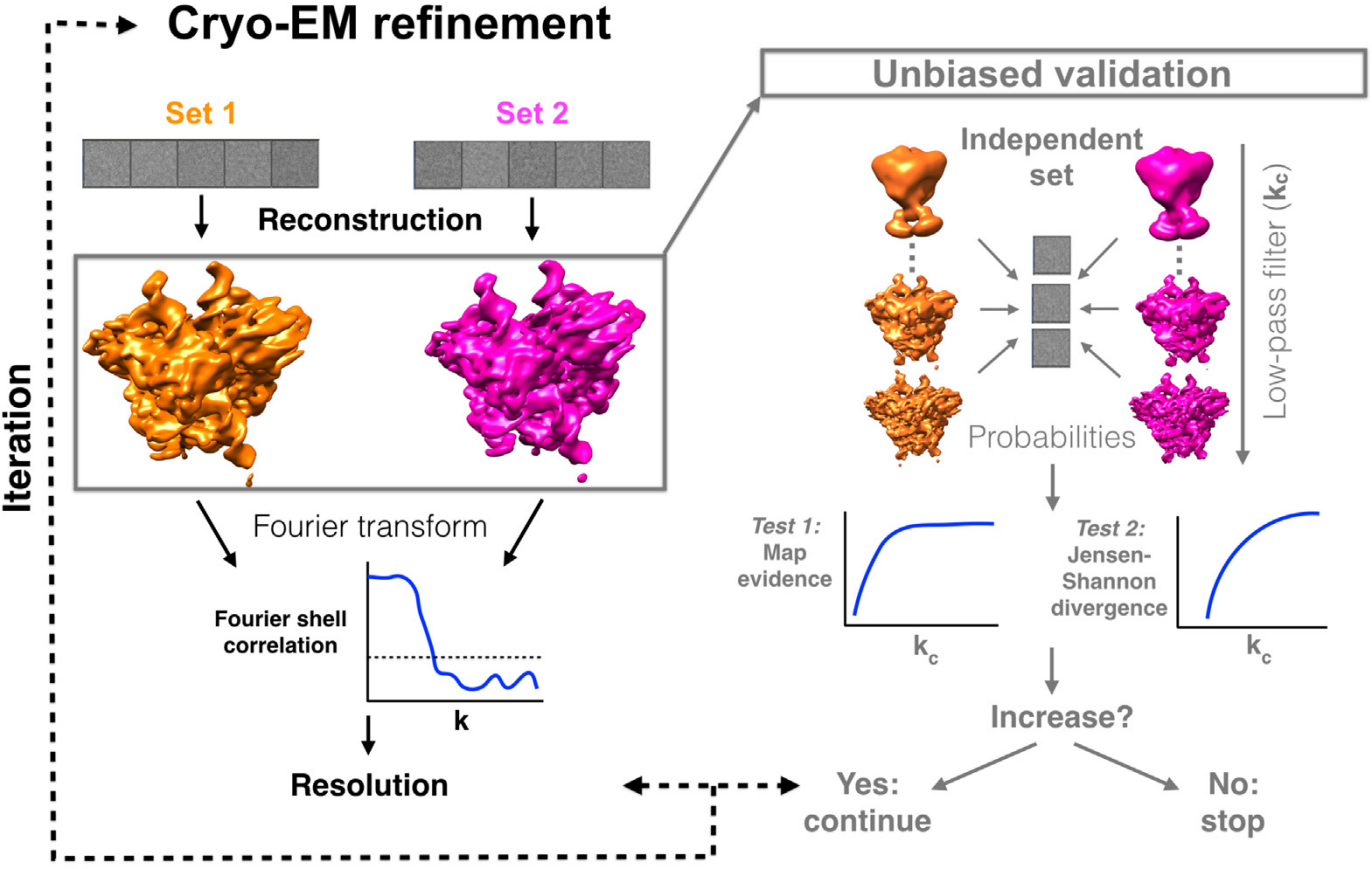
Unbiased validation protocol for cryo-EM maps using an independent particle set. (**left**) Gold-standard refinement procedure in cryo-EM. Two particle sets are used to generate two independent reconstructions. These reconstructions are compared using the Fourier shell correlation (FSC). A fixed FSC threshold is used to extract the resolution of the reconstructions. The process is iterated until the resolution stops improving. (**right**) Novel validation protocol using a small independent particle set not included in the gold-standard refinement. At each iteration of the refinement, the reconstructions are low-pass filtered to different frequency cutoffs $k_{c}$. The BioEM probabilities (Cossio and Hummer, 2013, Cossio et al., 2017), over the independent control set, are calculated as a function of $k_{c}$. Two tests validate the quality of the reconstructions: 1) the map evidence of the log-posterior and 2) the statistical similarity between the probability distributions (measured with a normalized Jensen-Shannon divergence). The results from both tests should increase as a function of the frequency cutoff. The maps represented correspond to the RAG1-RAG2 comple.x (see Methods).

Fig. 1 shows the work-flow of the methodology. The refinement is done following the gold-standard procedure (Fig. 1-left), where two reconstructions are generated at each iteration step. These two reconstructions are validated using the independent particle set (Fig. 1-right). At each iteration, the two 3D maps are low-pass filtered to different frequency cutoffs, $k_{c}$ (see Methods). The BioEM (Cossio and Hummer, 2013) probability, $P_{i\omega }(k_{c})$, is calculated for each gold-standard reconstruction, i=1,2, over the control set of images (ω) with *N* particles. As a first validation test, we monitor the map evidence, $\sum_{\omega }\ln(P_{i\omega }(k_{c}))/N$, as a function of $k_{c}$ for each set *i*. This evidence should increase or remain constant as higher frequencies are added to the maps. Failing this test is a prime indicative that there is a problem in the refinement process.

The second validation test consists on measuring the similarity between the probability distributions of the two reconstructions, also as a function of the frequency cutoff. For this purpose, we calculate a normalized Jensen-Shannon divergence (NJSD) (see Methods). The NJSD is a positive, symmetric and bound metric that measures how distinguishable are the probability distributions from the reconstructions sets 1 and 2. We expect that, as more frequencies are added to the reconstructions, more noise is added, and the densities of probability distributions are less similar.

In the following, we describe in detail the two validation tests.

### Map evidence from the log-posterior

We tested the methodology over several cryo-EM datasets: the synaptic RAG1-RAG2 complex (RAG1-RAG2) (Ru and Others, 2015), the human HCN1 channel (HCN1) (Lee and MacKinnon, 2017), and the TRPV1 ion channel (TRPV1) (Maofu et al., 2013). These systems represent a diverse set of biomolecular families, with membrane proteins and protein-nucleic acid complexes. The reconstruction refinement was performed using the gold-standard procedure in RELION (Scheres, 2012). The final resolution of these systems ranges from approximately 3 to 6 Å(see Methods). To analyze the impact of aligning noise, we studied two additional systems: cryo-EM reconstructions from the HIV-1 envelop trimer (HIV-ET) (Mao et al., 2013) and a set of synthetic pure-noise images that acts as a ‘false’ control set with the RAG1-RAG2 reconstructions (see Methods). This was motivated by the fact that some reconstructions might have been generated from pure-noise particles, and their resolution might have been over-estimated (Subramaniam, 2013, Henderson, 2013, Shatsky et al., 2009).

**Fig. 2:**
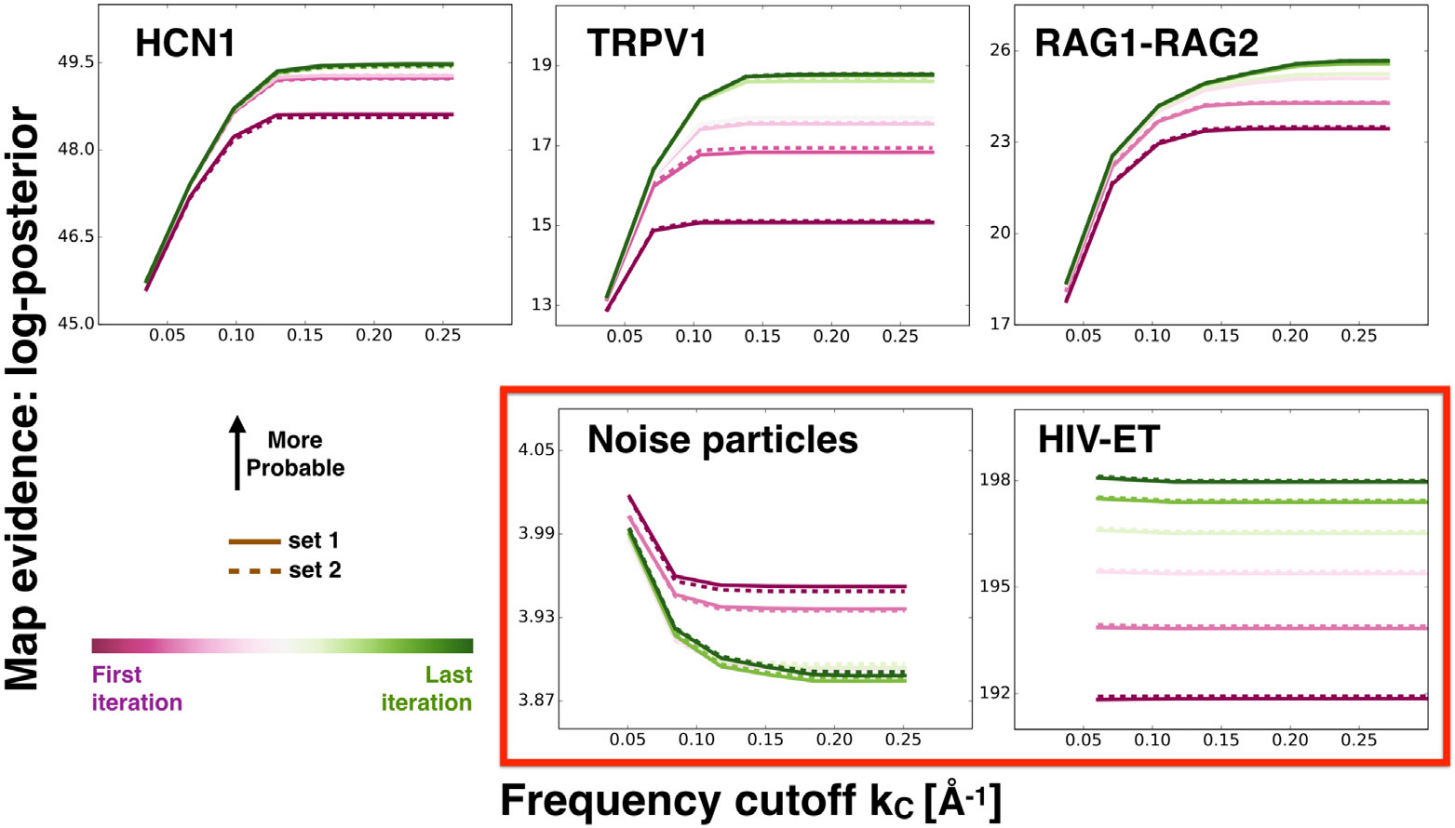
The sum of the log-posterior relative to noise $\sum_{\omega }\ln(P_{i\omega })/N-\ln(P_{\mathrm{Noise}})$, over the control set with *N* particles, as a function of the frequency cutoff for reconstructions from set i=1 and 2 (solid and dashed lines, respectively). The results are shown for different refinement iteration steps with a gradient color code: the first iteration is maroon and the last iteration is green. On the top row, we show the results for the cryo-EM systems where we expect refinement to work properly: HCN1, TRPV1 and RAG1-RAG2 for N=5000. Systems that present signs of treating noise as signal, the HIV-ET with N=5000 and a noise particle control set with N=1000, are shown in the bottom row, highlighted with a red box.

In Fig. 2, we examine the improvement of the maps by monitoring the sum of the log-posterior relative to noise $\sum_{\omega }\ln(P_{i\omega }(k_{c}))/N-\ln(P_{\mathrm{Noise}})$, over the control set with N=5000, as a function of $k_{c}$ for the reconstructions i=1,2. The results are shown for different refinement iterations with a gradient color scheme (first iteration: maroon; last iteration: green). These results measure how probable each filtered map is relative to $P_{\mathrm{Noise}}$. For the RAG1-RAG2, HCN1 and TRPV1 systems, we find an increase of the map evidence (given by the sum of the log-posterior) as a function of the frequency cutoff. For very high frequencies, the map evidence plateaus. We only observe minor differences between the results obtained for reconstructions i=1 and 2 (solid and dashed lines, respectively, in Fig. 2). This is an indication of the similarity between the two reconstructions. Importantly, the results highlight the ability of the BioEM posterior to correctly rank maps of different resolutions. For example, in Supplementary Fig. 1, we show the log-posterior for the first five iterations of the refinement of the TRPV1 system. Even for low-resolution maps, as the iteration increases (*i.e.*, the map resolution) so does the map evidence. The reconstructions from the last iterations (*i.e.*, the most refined with highest resolution) are the most probable. This is in agreement with what one expects from the 3D-refinement algorithms (Cossio and Hummer, 2018), and from previous studies that use the posterior probability to discriminate between conformational models (Cossio and Hummer, 2013, Cossio et al., 2018).

In contrast, for the HIV-ET and noise-particle set, we find a different behavior of the map evidence. We find that the sum of the log-posterior does not increase as a function of the frequency cutoff but decreases or remains constant. For the noise-particle set, the map evidence relative to $P_{\mathrm{Noise}}$ is small, and the differences between iterations are almost two orders of magnitude smaller than for the validated sets. Moreover, for this case, as the refinement iteration proceeds, the maps are slightly less probable over the control set. This analysis monitors map quality in cryo-EM: if the map evidence does not increase as a function of the frequency cutoff and the refinement iteration, then there are signs of spurious data in the refinement.

### Similarity between the probability distributions

As a second validation test, we compare the distributions of the posterior probabilities generated by the reconstructions from sets i=1,2 over the control set. In the Supplementary Information, we show an example of the probability distributions for the HCN1 system for two frequency cutoffs at a given iteration (Supplementary Fig. 2-top). We find that the probability distributions, over the independent set, are quite similar for both reconstructions. However, there are small differences between them, and the higher-frequency maps present larger fluctuations (Supplementary Fig. 2-bottom). These differences can be quantified using a normalized Jensen-Shannon divergence (NJSD; see Methods).

**Fig. 3:**
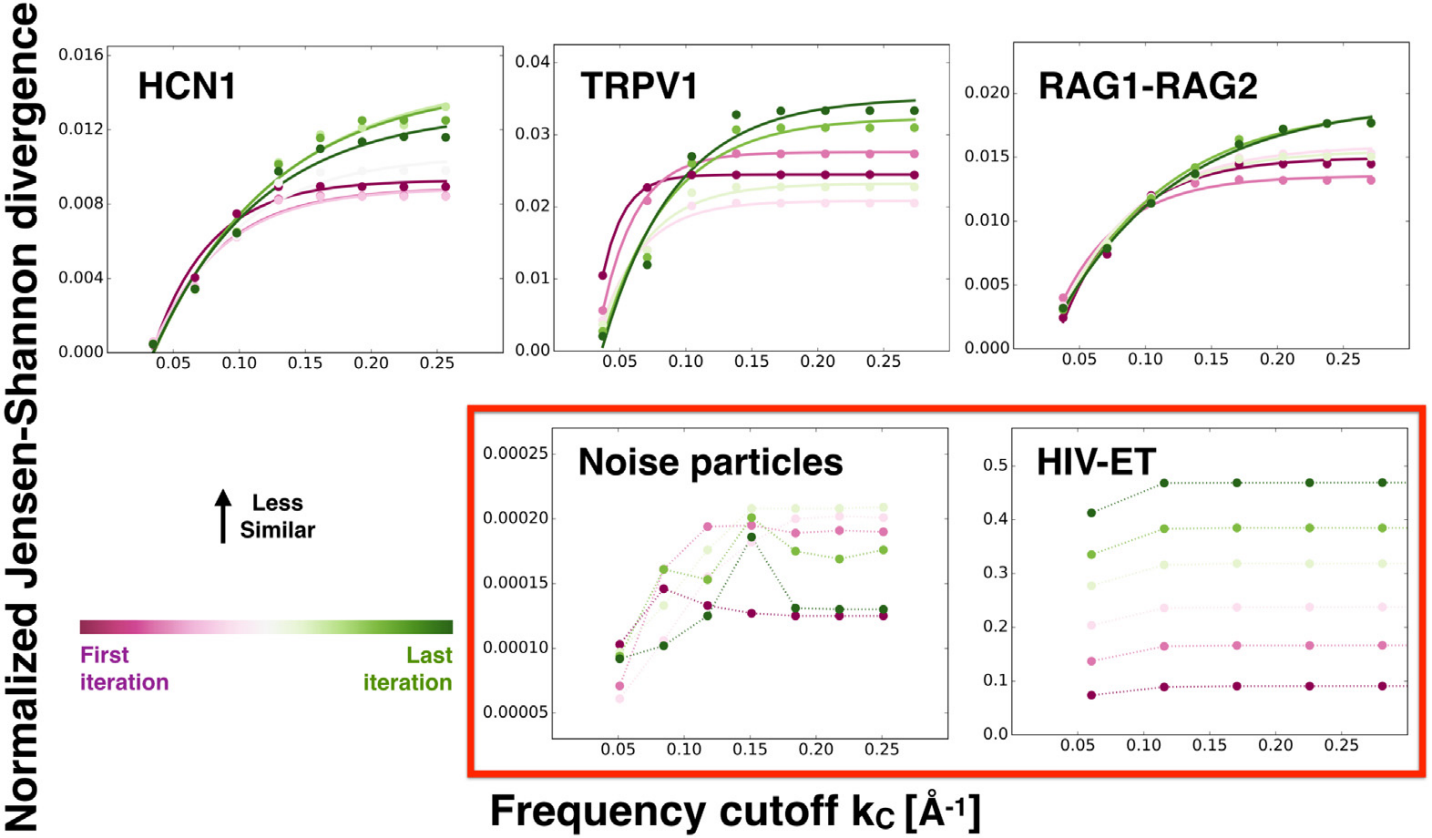
Normalized Jensen-Shannon divergence (NJSD) as a function of the frequency cutoff. This metric calculates the similarity between the distributions of the BioEM probabilities computed for the two reconstructions from sets 1 and 2. We use a gradient color code for the refinement iteration steps: the first iteration is maroon and the last iteration is green. On the top row, we show the results for the systems where standard cryo-EM refinement is expected to work: HCN1, TRPV1 and RAG1-RAG2. For these systems, we fit the data points to an inverse exponential function $-{\mathrm{Ae}}^{-k_{c}/\gamma }+B$ (solid lines). Systems that treat noise as signal due to the alignment, a noise particle control set and HIV-ET, are shown in the bottom row with the dashed lines as a guide, and highlighted by a red box. The number of particles in the control sets are the same as for the data in Fig. 2. (For interpretation of the references to color in this figure legend, the reader is referred to the web version of this article.)

In Fig. 3, we plot the NJSD as a function of the frequency cutoff $k_{c}$. Interestingly, for the RAG1-RAG2, HCN1 and TRPV1 systems, we observe that the NJSD increases as higher frequencies are included in the filtered maps. This implies that the probability distributions between maps with higher frequencies are less similar, possibly because they are more uncorrelated due to the high-frequency noise. For these standard systems, we also find that as the iteration increases the NJSD reaches at higher frequencies a plateau value. This behavior can be fitted with an inverse exponential function $-{\mathrm{Ae}}^{-k_{c}/\gamma }+B$ (see below and solid lines in Fig. 3). By contrast, for the HIV-ET and noise-particle sets, we find that the NJSD remains constant or has random behavior, suggesting that distributions do not consistently change when higher frequencies are added to the maps. For HIV-ET, we notice further that the NJSD values are ten times larger than for any of the other systems, indicating that the two gold-standard refinements are highly inconsistent with each other.

### Validation tests versus resolution

**Fig. 4:**
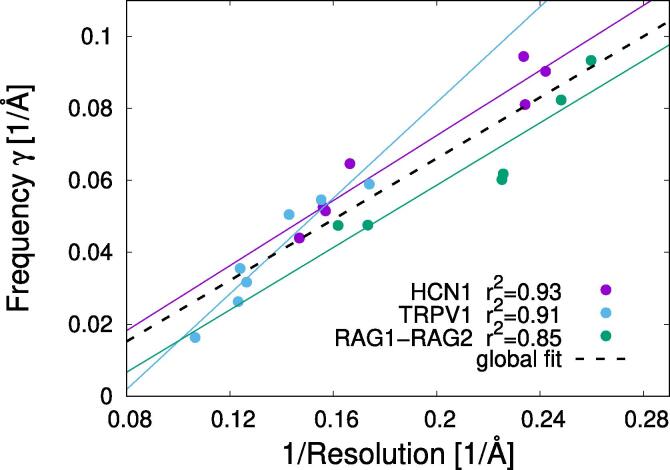
Frequency γ versus the inverse of the resolution for the standard cryo-EM systems: HCN1, TRPV1 and RAG1-RAG2. The NJSD curves for these systems were fitted to an inverse exponential function $-{\mathrm{Ae}}^{-k_{c}/\gamma }+B$ where γ is the frequency. We find large correlations between γ and the inverse of the resolution (calculated using the 0.143 criteria). The correlation coefficients are $r^{2}=0.93,0.91$, and 0.85, for HCN1, TRPV1 and RAG1-RAG2, respectively. Solid lines show the linear fits to the individual sets. Black dashed line shows the global fit with parameters γ=0.42/R-0.02 where *R* is the resolution. The correlation coefficient for the global fit is $r^{2}=0.86$.

We explored how the results depend on the map resolution. For the HCN1, TRPV1 and RAG1-RAG2 systems, we find that the NJSD curves can be fitted to an inverse exponential function, $-{\mathrm{Ae}}^{-k_{c}/\gamma }+B$ (solid lines shown in Fig. 3). Intuitively, the frequency γ indicates where the plateau of the NJSD is reached. In Fig. 3, we can qualitatively see that γ increases with each refinement iteration. In Fig. 4, we plot the frequency γ as a function of the inverse of the resolution (calculated using the FSC at the threshold 0.143). Interestingly, we find that the frequency γ is highly correlated to the inverse of the resolution with correlation coefficient $r^{2}=0.93,0.91$, and 0.85, for HCN1, TRPV1 and RAG1-RAG2, respectively. In Supplementary Fig. 3, we compare γ to the resolution obtained using the 1/2 bit non-fixed FSC threshold (Van Heel and Schatz, 2005), finding similar correlations. These results show that even from a small control set it is possible to extract unbiased information of the map resolution. We note that for the HIV-ET and noise-particle sets it is not possible to fit the NJSD data to an inverse exponential function. Therefore, we can only estimate the correlation between γ and the inverse of the resolution for the standard cryo-EM systems.

### Convergence over a small control set

We assessed how the results depend on the number of particles in the control set. In Supplementary Fig. 4, we show an example of the map evidence and NJSD as a function of the number of particles in the control set. We find that after approximately 1000 particles these observables converge, suggesting that only a small set is needed to perform the analysis. This is confirmed in Supplementary Fig. 5, where we plot the map evidence and NJSD as a function of the frequency cutoff for a validation set of 1000 particles. For the same set, in Supplementary Fig. 6, we plot the frequency γ as a function of the inverse of the map resolution, showing high correlation between the two for the standard cryo-EM systems. These results are very similar to those obtained for the validation set with 5000 particles.

## Conclusions

In this work, we have developed a novel methodology for validating cryo-EM reconstructions. The procedure is performed over a small particle set that is not used to generate the reconstructions. The independence of the particle set ensures that there are no biases due to the refinement in the validation.

Two validation tests are proposed to assess the performance of the maps over raw unbiased data. The first consists on monitoring the map evidence as a function of a low-pass filter frequency cutoff. The posterior should increase as a function of the frequency cutoff and the refinement iteration. In the second test, we assess the similarity between the probability distributions generated from the two reconstructions from the gold-standard procedure. The distributions should become less similar as higher frequencies are added to the reconstructions.

We performed the validation tests over several systems: three standard cryo-EM reconstruction sets, and two datasets that mimic the treatment of noise as signal. The results show substantial differences. While for the standard cryo-EM sets the results are as expected, the noisy datasets present almost no increment (and sometimes even decrease) of the map evidence or the NJSD. Thus, signatures of purious signal can be monitored by measuring the maps’ performance over an independent small set of particles. However, the method is still to be tested over more challenging datasets (not just those composed of mostly noisy particles). For example, for detecting the point at which noise is started to be treated as signal during the refinement.

The mathematical framework is not only valid for the BioEM posterior but also for any posterior probability that measures the likelihood of a 3D density given a particle set. However, an accurate estimate of the posterior probability is important. For example, if a large percentage of particles are misaligned then both the map evidence and NJSD will be underestimated (see Supplementary Fig. 7). With accurate posterior estimates, we find that the tests converge over a small particle set, typically only 1000 particles. We note that for the evaluated systems, the particle sets were selected from the EMPIAR dataset (Iudin et al., 2016) after 3D classification, which reduces the variability and heterogeneity of the particles. Therefore, the consequences of the particle picking and classification algorithms on the validation methodologies remain to be assessed.

The proposed methodology is still to be examined over flexible and heterogeneous biomolecules. However, preliminary tests using synthetic particles, which belonged to different conformations, showed that with the posterior probability, it is possible to determine the correct conformational ensemble (Cossio and Hummer, 2013). Moreover, novel reconstruction methods (Moscovich et al., 2019, Lederman et al., 2019, Zhong et al., 2019) that use machine learning techniques for mapping conformational variability will highly benefit from cross-validation procedures because for continuous heterogeneous ensembles the traditional FSC methods fail.

In summary, our methodology provides an unbiased basis to validate cryo-EM maps. Moreover, it has the potential to be applicable for directly validating atomic models (instead of 3D maps) using an independent set. We conclude that having a control particle set which is not tampered to generate reconstructions is beneficial for validating cryo-EM applications.

## Data availability

The BioEM code is available at https://github.com/bio-phys/BioEM. A tutorial to perform the cross-validation protocol is available at: https://github.com/bio-phys/BioEM-tutorials.

## Funding

S.O. and P.C. were supported by Colciencias, University of Antioquia and Ruta N, Colombia. G.H., and P.C. acknowledge the support of the Max Planck Society. Some computations were performed on a local server with an NVDIA Titan X GPU. PC gratefully acknowledges the support of NVIDIA Corporation for the donation of this GPU. Other computations were performed at the Max Planck Computing and Data Facility.

## Credit Author Statement

G.H. and P.C. conceived the idea. S.O. and P.C. developed the analysis and methodology. L.S. and M.R. developed and optimized the software. B. A. R validated the results. All authors wrote and reviewed the manuscript.

## Declaration of Competing Interest

The authors declare that they have no known competing financial interests or personal relationships that could have appeared to influence the work reported in this paper.
