# Supplementary material for: Validation tests for cryo-EM maps using an independent particle set
Source: J Struct Biol X. 2020 Jul 21;4:100032. doi: 10.1016/j.yjsbx.2020.100032 (PMC7385033; doi:10.1016/j.yjsbx.2020.100032)
Supplement: Supplementary data 1 [file mmc1.pdf]

Supplementary Information: Validation tests for cryo-EM maps using an independent particle set

Sebastian Ortiz[1], Luka Stanisic[2], Boris A Rodriguez[3], Markus Rampp[2], Gerhard Hummer[4,5], and Pilar Cossio[1,4,*]

[1] *Biophysics of Tropical Diseases, Max Planck Tandem Group, University of Antioquia UdeA, Calle 70 No. 52-21, Medellín, Colombia.*
[2] *Max Planck Computing and Data Facility, 85748 Garching, Germany.*
[3] *Grupo de Física Atómica y Molecular, Instituto de Física, Facultad de Ciencias Exactas y Naturales, Universidad de Antioquia UdeA, Calle 70 No. 52-21, Medellín, Colombia.*
[4] *Department of Theoretical Biophysics, Max Planck Institute of Biophysics, 60438 Frankfurt am Main, Germany.*
[5] *Institute of Biophysics, Goethe University, 60438 Frankfurt am Main, Germany.*
[*] *email:* pilar.cossio@biophys.mpg.de; grupotandem.biotd@udea.edu.co

Supplementary Figures

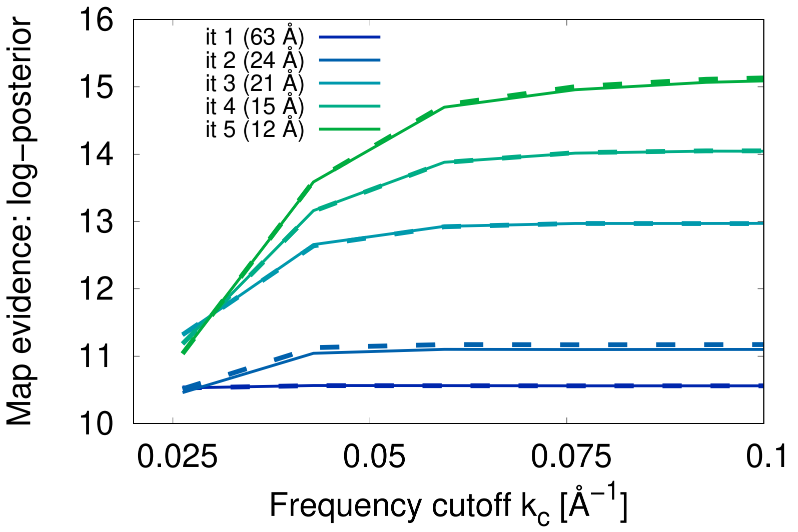

Supplementary Figure 1: *Map evidence for the first five refinement iterations of the TRPV1 system as a function of the frequency cutoff* k_c*.* The log-posterior is shown for the reconstructions from set 1 and set 2 with solid and dashed lines, respectively. The reconstruction's resolution for each iteration is presented in the label. The log-posterior increases as a function of the iteration step and frequency cutoff (apart for the reconstruction with lowest resolution of 63 Å). Note that because we are analyzing low-resolution reconstructions the k_c range is smaller than for main text Fig. 2.

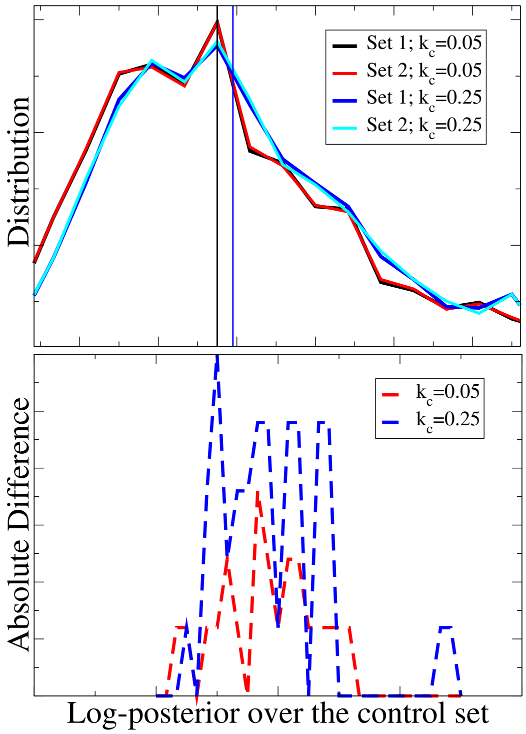

Supplementary Figure 2: *Differences in the log-posterior distributions.* (**top**) Examples of the distributions of the log-posterior relative to noise over the independent particle set. The distributions are calculated for the reconstructions from set 1 and set 2 at two cutoff frequencies $k_c = 0.05$ and 0.25 Å^{-1} for the fifth iteration of refinement of the HCN1 system. The vertical lines are the averages of the distributions. (**bottom**) Absolute value of the difference between the probability distributions from set 1 and set 2 for $k_c = 0.05$ and 0.25 Å^{-1}. The distributions calculated for the maps with higher frequencies are less similar.

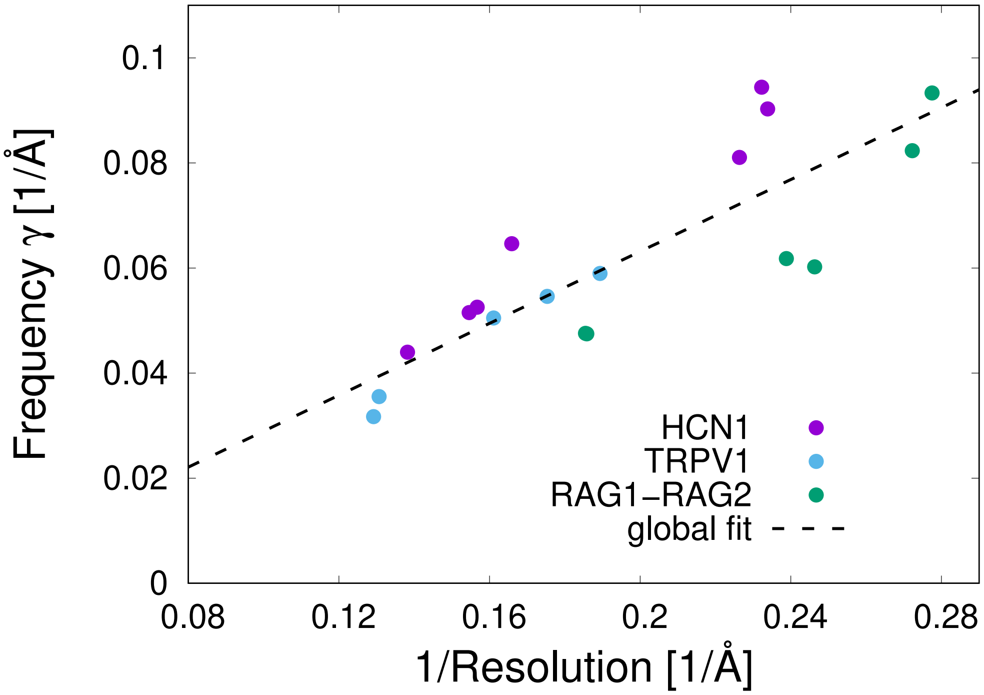

Supplementary Figure 3: *Frequency (γ) versus the inverse of the resolution using the 1/2 bit non-fixed FSC threshold (Van Heel, and Schatz, M. JSB 2005).* The results are shown for the standard cryo-EM systems: HCN1, TRPV1 and RAG1-RAG2. The correlation coefficients are $r^2 = 0.95$, 0.97, and 0.86, respectively. The dashed line shows the global linear fit with parameters $\gamma = 0.34/R - 0.005$ where R is the resolution. The FSC curves and resolution values were calculated using the webpage www.ImageScience.de/fsc. We note that for some of the low-resolution maps it was not possible to obtain a reliable FSC curve using this program, therefore, these points were omitted from the plot.

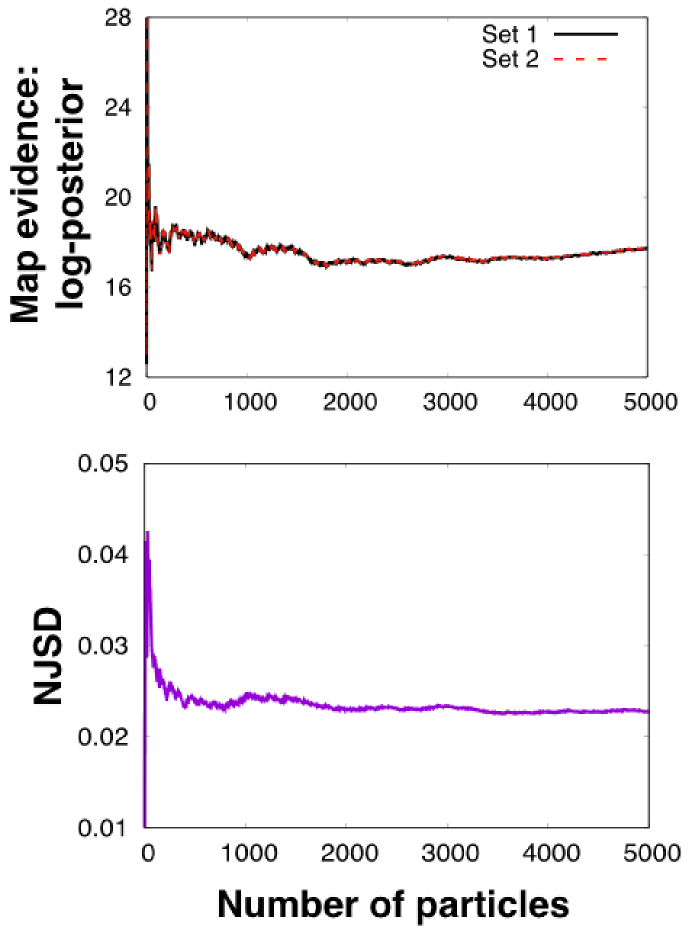

Supplementary Figure 4: *Convergence of the observables.* (**top**) The sum of the log-posterior relative to noise $\sum_\omega \ln(P_{i\omega})/N_\omega - \ln(P_{\text{Noise}})$ for set $i = 1$ and 2 (solid and dashed lines, respectively), and (**bottom**) the normalized Jensen-Shannon divergence as a function of the number of particles in the control set. The results are shown for the TRPV1 system for iteration 12 and cutoff frequency $k_c = 0.21$ Å^{-1}. The observables converge if more than approximately 1000 particles are used.

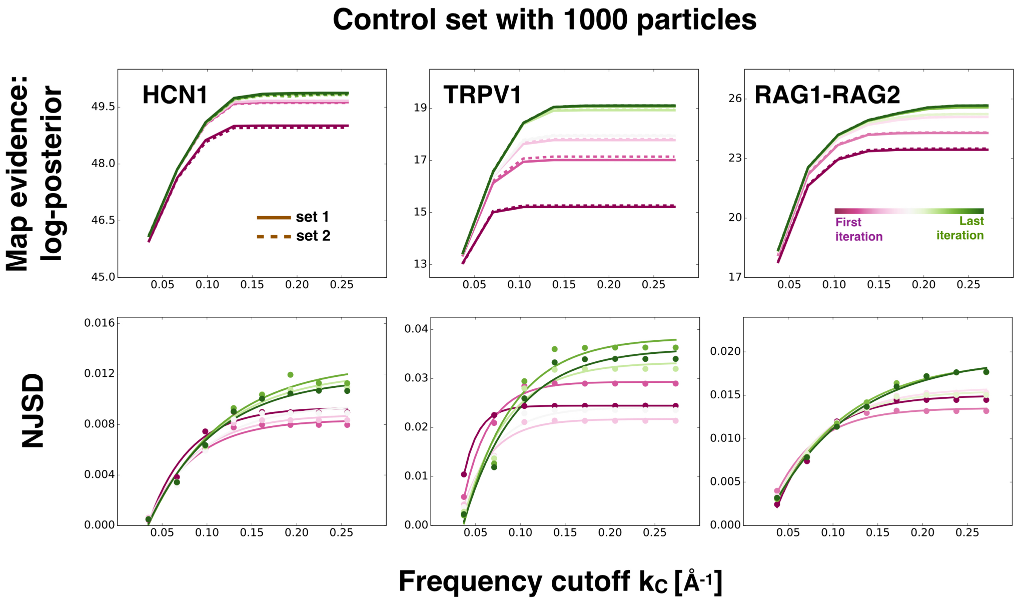

Supplementary Figure 5: *Map evidence and NJSD for a control set with 1000 particles.* (**top**) The sum of the log-posterior relative to noise and (**bottom**) the normalized Jensen-Shannon divergence as a function of the frequency cutoff. We use a gradient color code for the refinement iteration steps: the first iteration is maroon and the last iteration is green. The results are shown for the standard cryo-EM systems: HCN1, TRPV1 and RAG1-RAG2. The map evidence is shown for the reconstructions from set 1 as solid lines and set 2 as dashed lines. NSJD data is fit to an inverse exponential function $-Ae^{-k_c/\gamma} + B$ (solid lines; bottom).

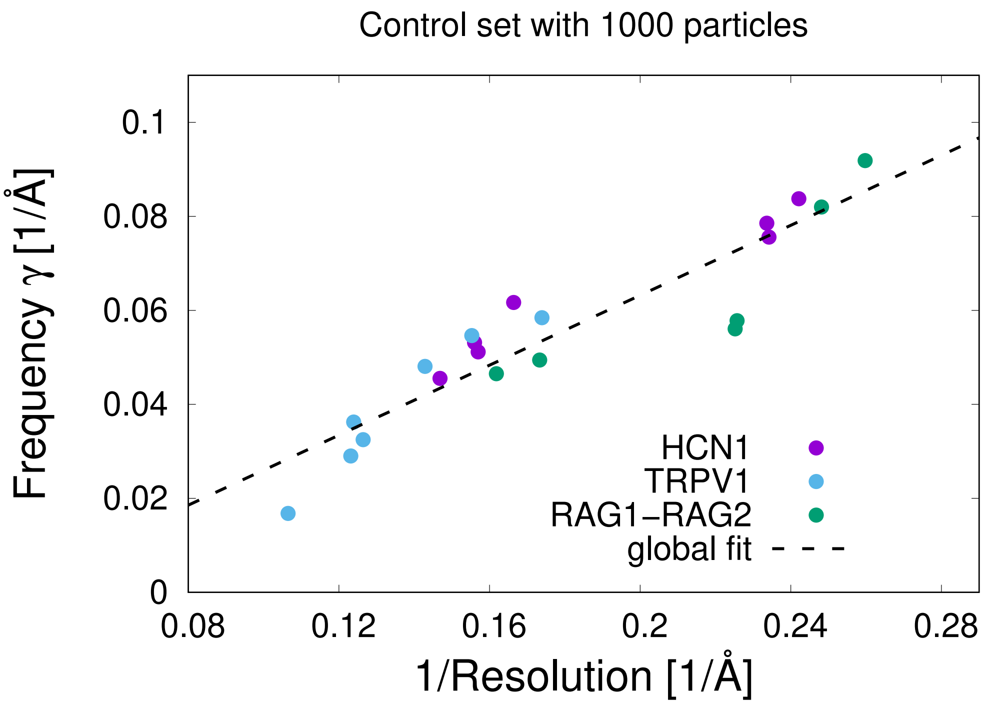

Supplementary Figure 6: *Frequency (γ) versus the inverse of the resolution for a control set with 1000 particles.* The results are shown for the standard cryo-EM systems: HCN1, TRPV1 and RAG1-RAG2. The correlation coefficients are $r^2 = 0.95$, 0.93, and 0.78, respectively. The dashed line shows the global linear fit with parameters $\gamma = 0.37/R - 0.01$ where R is the resolution. The resolution was estimated using the 0.143 FSC threshold with RELION.

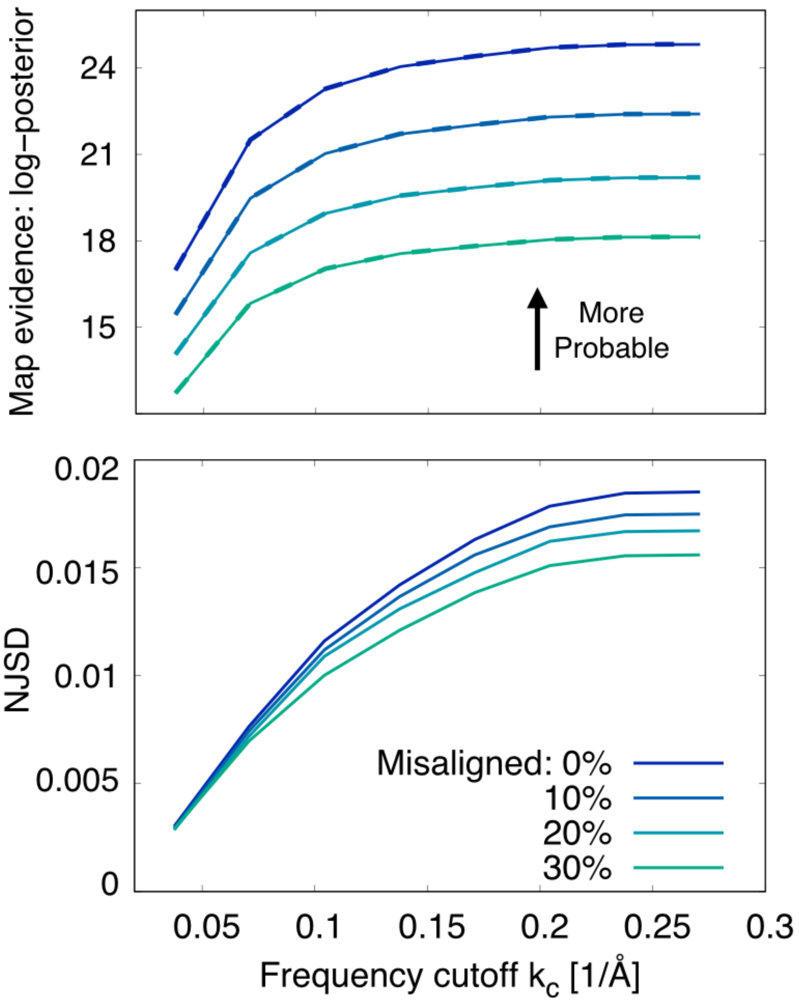

Supplementary Figure 7: *Map evidence (top) and NJSD (bottom) for percentages of misaligned particles.* The results are for the 8th iteration of refinement of the RAG1-RAG2 system for 0, 10, 20 and 30% of misaligned particles. The map evidence (top) is shown for the reconstructions from set 1 and set 2 with solid and dashed lines, respectively. The misalignment was performed by assigning random orientations to the list of quaternions, used in round 2, instead of the best orientations determined from BioEM round 1. Both the map evidence and the NJSD decrease as a larger percentage of particles are misaligned, indicating that the orientation alignment is important for an accurate estimate of the posterior probability.

Supplementary Text

BioEM input file examples

Round 1: Example of the BioEM input file for the TRPV1 system for round 1. The best orientations for each particle are obtained using the final map from the refinement. The following input file is for a subset of particles that have experimental defocus between 1.3 and 1.7 μm. The best 10 orientations for each particle are selected.

```
PIXEL_SIZE 1.22
NUMBER_PIXELS 256
USE_QUATERNIONS
CTF_DEFOCUS 1.3 1.7 10
CTF_B_ENV 0 10 2
CTF_AMPLITUDE 0.1 0.1 1
PRIOR_DEFOCUS_CENTER 1.5
SIGMA_PRIOR_DEFOCUS 0.8
SIGMA_PRIOR_B_CTF 1
DISPLACE_CENTER 30 1
WRITE_PROB_ANGLES 10
```

Round 2: Example of the BioEM input file for the TRPV1 system for round 2. The input file is for a single particle that has an experimental defocus of 1.9 μm.

```
PIXEL_SIZE 1.22
NUMBER_PIXELS 256
USE_QUATERNIONS
CTF_DEFOCUS 1.9 1.9 1
CTF_B_ENV 0 10 2
CTF_AMPLITUDE 0.1 0.1 1
PRIOR_DEFOCUS_CENTER 1.9
SIGMA_PRIOR_DEFOCUS 0.3
SIGMA_PRIOR_B_CTF 1
DISPLACE_CENTER 30 1
```

Pure-noise particles

We generated a set of 1000 synthetic pure-noise particles. Each particle has an image size of 180×180 and a pixel size of 1.23 Å. The particles contain random intensities following a Gaussian distribution with zero mean and unit variance. Because there is no experimental defocus, the BioEM probabilities are computed by performing round 1 with defocus range between 0.5 and 4.5 μm and using 4608 quaternions uniformly distributed in orientation space. This analysis was performed for each of the refined maps of the RAG1-RAG2 system.
